# How Big Data Affect Urban Low-Carbon Transformation—A Quasi-Natural Experiment from China

**DOI:** 10.3390/ijerph192316351

**Published:** 2022-12-06

**Authors:** Ning Xu, He Zhang, Tixin Li, Xiao Ling, Qian Shen

**Affiliations:** 1 School of Political Science and Public Administration, Henan Normal University, Xinxiang 453007, China; 2 State Information Center, Beijing 100045, China; 3 School of Economics and Management, Kunming University, Kunming 650214, China; 4 School of Business, Hubei University, Wuhan 430062, China; 5 Finance Department, Guangdong University of Finance & Economics, Guangzhou 510320, China

**Keywords:** national big data comprehensive pilot zone, urban low-carbon transformation, difference-in-differences model, green innovation, resource elements allocation, spatial spillover effect

## Abstract

As a new factor of production, data play a key role in driving low-carbon and sustainable development relying on the digital economy. However, previous studies have ignored this point. Based on the panel data of 283 cities in China from 2007 to 2019, we investigated the construction of national big data comprehensive pilot zones (NBDCPZs) in China as a quasi-natural experiment, using the difference-in-differences (DID) model to empirically test the impact of NBDCPZ policies on urban low-carbon transformation. The following conclusions can be drawn: NBDCPZ construction significantly promotes urban low-carbon transformation, and a series of robustness analysis supports this conclusion. NBDCPZ constructions mainly promotes urban low-carbon transformation by stimulating urban green innovation and optimizing the allocation of urban resource elements. Compared with eastern cities, small and medium-sized cities, and resource-based cities, the construction of NBDCPZs can promote the low-carbon transformation of cities in central and western China, large cities, and non-resource-based cities. Further analysis shows that the construction of NBDCPZs can only improve the low-carbon transformation of local cities, with negative spatial spillover effects on the low-carbon transformation of surrounding cities. Therefore, in the future, it is vital to consider the promotion effect of the construction of NBDCPZs on the low-carbon transformation of local cities and prevent its negative impact on the low-carbon transformation of surrounding cities.

## 1. Introduction

Global warming is a serious threat to the human living environment. Carbon dioxide emissions and other pollution indicators (such as SO_2_ and nitrogen oxides) are the main “culprits” of global warming [1]. The development of a low-carbon economy is considered an effective way to cope with global warming. As an important participant in tackling global warming, China solemnly promises to achieve a carbon peak by 2030 and carbon neutrality by 2060. This “double carbon” goal not only points out the direction but also imposes increased requirements for China’s low-carbon economic transformation. In particular, in the context of the COVID-19 pandemic, it is urgent to explore new drivers of China’s low-carbon development. Furthermore, the digital economy based on 5G, big data, artificial intelligence, the Internet of Things, and other next-generation information technologies is profoundly changing the means of production and social governance [2]. The digital economy is a series of economic activities involving digital technology, digital equipment, digital knowledge, and information as production factors [3,4]. The digital economy is the most dynamic, innovative, shared, and resilient economic form in the context of the current global epidemic. Data are the core element involved in the development of the digital economy, and governments around the world are actively developing digital economies with the data element as the core. For example, the United States formulated the Big Data Research and Development Plan in 2011 [5], and the EU issued the EU Data Strategy in 2020 [6]. In order to promote the development of China’s digital economy, the Chinese government wrote big data into a government work report for the first time in 2014, and the Plan of Action for Promoting Big Data Development issued in 2015 has identified big data as a national strategy. The report of the 19th National Congress of the Communist Party of China in 2017 required the promotion and deep integration of big data and the real economy. In 2020, the Chinese government identified data as a factor of production, and in 2021, China’s 14th Five Year Plan set the improvement of a big data standard system as the focus of development. A series of policies fully demonstrates that big data have become the key element and the new driving factor of China’s economic transformation and development. With the development of big data, China’s digital economy accounted for 39.8% of GDP in 2021. In order to accelerate the deep development and application of big data and summarize and refine the successful development experience of the big data industry, the Chinese government approved Guizhou Province as the first national big data comprehensive pilot zone (NBDCPZ) in 2015. In 2016, the second batch of comprehensive pilot areas was approved in nine provinces (cities), including Beijing. For example, there have been more than 5000 big data enterprises established in Gui’an City since 2021. The revenue of software and information technology services increased by 67.74%, reaching RMB 38.8 billion, accounting for 91.04% of the province’s total, with the digital economy accounting for 41.9% of the region’s GDP. In addition, the bandwidth of Gui’an’s national Internet backbone direct connection point was expanded to 500 G, with a direct connection with 28 cities, including Beijing, Shanghai, Guangzhou, and Shenzhen (https://www.guiyang.gov.cn/zwgk/zwgkxwdt/zwgkxwdtbmdt/202204/t20220427_73651156.html, accessed on 4 December 2022). Under the dual background of “dual carbon” and digital economy, can the construction of NBDCPZs promote the low-carbon transformation of China’s economy? If the answer is yes, what is the impact mechanism? What are the characteristics of impact heterogeneity? Is there any spatial spillover effect? Studying these issues will help the Chinese government clarify the relationship between the digital economy and low-carbon development, with China’s theoretical and practical experience serving as an example for the world to better use the digital economy to support low-carbon and sustainable development.

With the global warming caused by carbon dioxide becoming increasingly serious, some researchers began to use carbon emission intensity [7,8], carbon emission efficiency, or carbon emission performance [9,10,11] to evaluate the low-carbon transformation of the economy. Other researchers have considered the influencing factors of low-carbon economic transformation from the perspectives of economic spatial agglomeration [12], green innovation [13], global value-chain embeddedness [14], urbanization [15], low-carbon policy [16], innovation policy [17], etc. In addition, a number of studies have focused on the green and low-carbon effects of the digital economy. Most scholars believe that the development of the digital economy can effectively replace traditional factors with data, help reduce environmental pollution through technological innovation [18,19], and reduce carbon emission intensity by optimizing industrial structure [20]. However, some researchers suggest that the application of digital equipment and infrastructure has increased energy demand [21,22], which is not conducive to green and low-carbon development. Some of the literature also indicates that the development of the digital economy and high-quality green development have a positive, nonlinear connection [23]. As far as specific carbon emission performance and efficiency are concerned, most studies include an indicator system to evaluate the digital economy and examine its effect on low-carbon transformation from the perspectives of artificial intelligence [24], digital inclusive finance [25], and Internet development [26,27,28]. Some studies consider “Broadband China” as a quasi-natural experiment, indicating that the impact of Internet development on energy conservation and emission reduction efficiency is non-linear [29]. In the era of the digital economy, data have no specific value, but digital and intelligent data elements have unique value [30]. The existing literature includes multilevel discussions on data elements from the perspectives of their connotation [30], characteristics [31,32], market cultivation mechanism [33], value creation [34], etc., as well as examinations of their enabling effect on traditional production factors [35], innovation effect [36], and economic growth effect [37,38]. Researchers employed the text analysis method to examine the impact of data elements on the high-quality development of the manufacturing industry [39]. With the gradual development of the event analysis method, researchers who combined it with theoretical and empirical analysis concluded that NBDCPZs can promote the digital transformation of enterprises by improving the construction of digital infrastructure, promoting the development of digital industry, and improving the intensity of financial science and technology expenditure [40], in addition to helping improve air pollution by promoting industrial upgrading and technological innovation and optimizing resource allocation [41]. The low-carbon transformation effect of the construction of NBDCPZs remains to be further studied.

The above research has laid a foundation for in-depth understanding of the green and low-carbon effects of the digital economy. However, the following shortcomings remain. On the one hand, data elements are the core content and key driving force of the digital economy, and although many studies have examined the green and low-carbon effects of the digital economy from the perspectives of artificial intelligence, digital inclusive finance, Internet development, etc., few studies focus on the impact of big data on the low-carbon transformation of the economy. On the other hand, most studies use an indicator system to evaluate the digital economy, examining its low-carbon transformation effect and ignoring the endogenous problem between the two, which may underestimate the low-carbon transformation effect of the digital economy. In contrast to existing research, in this study, we focus on the urban level from the perspective of data production factors, with NBDCPZs as a quasi-natural experiment and a deep commitment to research on the impact of big data on urban low-carbon transformation and development. The possible marginal contributions of this study are mainly reflected in the following three aspects. (1) This study comprises research from the perspective of big data, with thorough discussion of the contribution and impact mechanism of big data in promoting urban low-carbon development, with a review of relevant literature on the digital economy and the low-carbon economy. (2) We considered the “quasi-natural experiment” of NBDCPZs, alleviating the endogenous problem of using an indicator system to evaluate the low-carbon transformation effect of the digital economy. (3) We further investigated the heterogeneity and spatial spillover effects of NBDCPZ construction on urban low-carbon transformation, contributing to the understanding that big data affect urban low-carbon transformation.

## 2. Theoretical Mechanism and Research Hypothesis

Emission reduction and efficiency enhancement is the most direct embodiment of urban low-carbon transformation. On the one hand, data elements and traditional production factors such as labor, capital, and energy complement and cooperate with one another and combine to activate traditional production factors and improve total factor productivity [42]. On the other hand, big data can achieve more accurate monitoring, analysis, prediction, and early warning of carbon emissions by establishing an environmental monitoring data center, improving the pertinence, scientificity, and timeliness of environmental regulatory decisions, thus providing strong support for precise and scientific emissions reduction [43]. Although China has made some progress in the development and application of big data, problems such as insufficient data opening and sharing, weak digital infrastructure, and imperfect systems and mechanisms persist [5]. Open data resource management and sharing, data center integration, data resource application, data element circulation, big data industry agglomeration, big data international cooperation, and big data system innovation are the seven major tasks of NBDCPZ construction [5]. With these seven tasks, NBDCPZs can break data resource barriers; strengthen digital infrastructure construction; formulate laws, regulations, and standards; and highlight the low-carbon transformation effect of big data.

The construction of NBDCPZs can also promote urban green innovation. The application of big data can generate new business forms such as digital platforms and digital finance so as to achieve deeper integration with traditional industries [44]. Networked collaboration between industries can expand the exchange and sharing of data and knowledge elements, which is conducive to urban innovation. The medium of information exchange is one of the main applications of big data, which can effectively reduce the information asymmetry between the needs of enterprises and consumers, thus facilitating the collaborative evolution between enterprises and consumers and playing an important role in promoting the construction of a market demand-oriented green innovation mode [45]. The construction of NBDCPZs can reduce the cost of information infrastructure and accelerate the process of enterprise green innovation, which is conducive to promoting urban green innovation [46]. In addition, NBDCPZ construction optimizes the process of green innovation activities of enterprises by integrating big data technology and big data thinking into green innovation decisions. The construction of NBDCPZs is conducive to improving the allocation of urban resources. Big data construction can improve the matching of the degree of labor demand and supply, reshaping and improving the form and efficiency of labor resource allocation [47]. With the help of NBDCPZ construction, data elements reduce transaction costs and investment risks in the capital market, optimize the investment structure, and guide the allocation of funds to green and low-carbon industries. In addition, the construction of NBDCPZs can help improve the infrastructure of energy big data, integrate various types of energy consumption data of enterprises and residents, and accurately allocate energy elements from the supply side and the demand side. 

Based on above analysis, in this study, we propose the following hypotheses:

**Hypothesis** **1** **(H1).** *The construction of NBDCPZs is conducive to promoting urban low-carbon transformation.*

**Hypothesis** **2** **(H2).** *The construction of NBDCPZs can promote urban low-carbon transformation through green innovation and resource element allocation.*

NBDCPZ construction can also affect urban low-carbon transformation through spatial spillover effects. First, the construction of NBDCPZs is conducive to the formation of digital industry clusters, promoting the spread of green technologies and green processes in NBDCPZs, causing spatial spillover of green technologies. Secondly, the construction of NBDCPZs is conducive to the joint prevention and control of regional pollution and the realization of regional coordinated emissions reduction. On the one hand, NBDCPZ construction encourages enterprises in the pilot areas and surrounding cities to optimize production processes and achieve cleaner production by sharing and jointly building environmental pollution treatment facilities. On the other hand, the construction of NBDCPZs is conducive to realizing the interconnection and sharing of environmental monitoring data, thereby reducing environmental governance costs caused by information asymmetry [48] and promoting resource conservation and pollution reduction in adjacent areas. Finally, during the period of NBDCPZ construction, both local and surrounding cities are faced with the process of factor restructuring, especially when a cluster of various factors forms in the pilot areas driven by policies, causing a “siphon effect” on the surrounding areas. Such an effect causes the surrounding areas to only retain the original production links, maintaining traditional production mode and suppressing the green transformation of the surrounding areas with “negative” spillovers. Moreover, knowledge and technology spillovers of NBDCPZ construction may not play a positive role, and some failed practices and experiences may lead the surrounding areas to go astray in the continuous imitation and learning. Accordingly, in this study, we propose the following hypotheses:

**Hypothesis** **3** **(H3).** *The construction of NBDCPZs can produce a spatial spillover effect on urban low-carbon transformation.*

## 3. Empirical Design

### 3.1. Model Setting

In order to identify the impact of NBDCPZ construction on urban low-carbon transformation, in this study, we used a two-way fixed effect benchmark regression model based on the difference-in-differences (DID) method to evaluate the low-carbon transformation effect of NBDCPZ pilot policies. The benchmark regression model is as follows:

(1) $$Eff_{it}=\alpha_{0}+\alpha_{1}Policy_{it}+\varphi X_{it}+\mu_{i}+v_{t}+\varepsilon_{it}$$

where *Eff_it_* refers to the low-carbon transformation level of city *i* in year *t*, *Policy_it_* is the pilot variable of NBDCPZ policy, *X_it_* is a series of control variables that affect the low-carbon transformation of cities, $\mu_{i}$ represents the individual fixed effect, $v_{t}$ represents the time fixed effect, and $\varepsilon_{it}$ represents the random interference term. In order to investigate the two conduction mechanisms of green innovation (*INNOV*) and resource allocation (*AE*) of NBDCPZ construction that affect urban low-carbon transformation, in this study, we referred to the panel intermediary effect proposed by Baron a Kenny [49] and built models (2) and (3) on the basis of model (1):

(2) $$Med_{it}=\beta_{0}+\beta_{1}Policy_{it}+\varphi X_{it}+\mu_{i}+v_{t}+\varepsilon_{it}$$

(3) $$Eff_{it}=\gamma_{0}+\gamma_{1}Policy+\gamma_{2}Med_{it}+\varphi X_{it}+\mu_{i}+v_{t}+\varepsilon_{it}$$

In model (2) and model (3), *Med* is the intermediary variable, representing green innovation and resource element allocation, $\beta_{1}$ is the impact coefficient of NBDCPZ policy pilot variables on intermediary variables, and $\gamma_{2\;}$ is the impact coefficient of intermediary variables on urban low-carbon transformation. If $\beta_{1\;}$ and $\gamma_{2\;}$ are significant, the construction of NBDCPZs can affect urban low-carbon transformation through intermediary variables. At this time, if $\gamma_{1\;}$ is still significant but smaller than the coefficient $\alpha_{1}$ in the benchmark model (1), there is a partial mediation effect in the influence mechanism; if coefficient $\gamma_{1\;}$ is not significant, there is a complete mediation effect. The meaning of other variables is consistent with model (1).

### 3.2. Variable Selection

#### 3.2.1. Explained Variable: Urban Low-Carbon Transformation 

Carbon emission efficiency refers to both carbon emission reduction and economic efficiency [50], as well as the ability to achieve the maximum economic output with the minimum element input and minimum carbon emissions. Therefore, we used urban carbon emission efficiency to characterize urban low-carbon transformation. The measurement of urban carbon emission efficiency involves input and output indicators. We selected specific indicators with reference to existing studies [51]. The input indicators include labor force and capital. The labor force input is measured by total urban employment [51,52], and capital input is measured by the urban capital stock based on the perpetual inventory method [51,53]. Output indicators include expected output and unexpected output, and urban GDP is used to measure urban expected output [51]. With reference to the IPCC method (2006), the city’s unexpected output is measured by the carbon emissions generated by the consumption of natural gas, liquefied petroleum gas, electricity, and heat energy in the whole city [28]. Combined with the research method proposed by Tone and Tsutsui [54] we used the super efficiency EBM model to calculate the urban carbon emission efficiency.

#### 3.2.2. Explanatory Variable: NBDCPZ Policies 

During the sample period, we assigned the approved pilot cities (experimental groups) of NBDCPZ as 1 and the unapproved non-pilot cities (control groups) as 0, which is recorded as “*Treated_i_*”. In the pilot cities, weed 1 to the year when the pilot city was approved (2015 or 2016) and later years and 0 to the other years. The year corresponding to non-pilot cities was always assigned as 0. The year was recorded as “*Time_t_*”. In this case, *Policy_it_* = *Treated_i_* × *Time_t_*.

#### 3.2.3. Control Variable 

In this study, other factors that may affect the low-carbon transformation of cities were also included in the DID model so as to mitigate the error of missing variables to the greatest extent possible.

(1) Economic development level (*AGDP*): The higher the *AGDP*, the more intensive and effective the economic development model and the stronger the city’s ability to reduce emissions and improve efficiency. We used logarithmic GDP per capita to measure *AGDP* [51].
(2) Urbanization (*URB*): The higher the level of *URB*, the more compact urban production and life, which can promote urban centralized pollution control and efficient production. We used the proportion of permanent population in the total population at the end of the year to measure the *URB* [55].
(3) Environmental regulation (*ER*): *ER* can force enterprises to carry out green innovation and promote urban low-carbon transformation. It may also increase enterprise costs, which is not conducive to urban low-carbon transformation. In this study, three indicators, namely the sulfur dioxide removal rate, the industrial smoke (dust) removal rate, and the industrial solid waste comprehensive utilization rate, were selected to measure *ER* using the comprehensive index synthesized by entropy method [28].
(4) Population agglomeration (*AGG*): *AGG* can produce economies of scale and technology spillover effects to promote urban low-carbon transformation [56], but excessive *AGG* causes environmental pollution and aggravates carbon emissions, which is unfavorable to urban low-carbon transformation. We used population density to measure *AGG* [51].
(5) Openness (*OPEN*): *OPEN* can bring mature management experience and advanced pollution control technology for urban low-carbon transformation. On the other hand, it may bring overcapacity and backward, high-pollution industries into the home country. We used the proportion of foreign direct investment in GDP to measure *OPEN*.
(6) Traffic infrastructure (*ROAD*): A high level of *ROAD* helps to reduce urban traffic congestion and slow down urban carbon emissions. We used per capita road area to measure *ROAD*.
(7) Fiscal decentralization (*FIS*): *FIS* increases the ability of local governments to intervene in economic development, helping to reduce pollution and emissions according to the requirements of local residents with respect to environmental pollution. In order to assess political performance and maximize economic benefits, local governments may encourage the development of industries with a high tax base, causing pollution problems, which are unfavorable to the low-carbon transformation of cities. We used fiscal expenditure decentralization (the proportion of the city’s per capita fiscal expenditure relative to the sum of the central and local per capita fiscal expenditure) to measure *FIS*.
(8) Financial development (*FIN*): *FIN* can ease the financing constraints of enterprises and promote technological innovation of enterprises, which is conducive to the low-carbon transformation of cities. On the other hand, it may also increase the capital and polluting project investment of enterprises, which is not conducive to the low-carbon transformation of cities. We used the proportion of year-end loan balance in relative to GDP to measure *FIN*.

#### 3.2.4. Mediating Variable 

We measured the green innovation effect (*INNOV*) as the number of green invention patent applications per 10000 persons in the city [57,58]. With respect to resource element allocation (*AE*), with reference to existing research [59], we adopted the transcendental logarithmic Cobb–Douglas production function to calculate resource element allocation efficiency (the sum of labor, capital, and energy element allocation efficiency) of 283 cities in each year.

### 3.3. Data Source and Description

Owing to the serious lack of data in some Chinese cities, such as Zhongwei City, Lhasa City, Bijie City, Tongren City, Chaohu City, Sansha City, Sanya City, and Hegang City, we did not include all Chinese cities as research objects. We selected a set of panel data for 283 cities in China as the research object, with the research period spanning 2007 to 2019. The original data variables are from *China Urban Statistical Yearbook*, the *China Energy Statistical Yearbook*, provincial and municipal statistical yearbooks, and the EPS data platform. Linear interpolation was used to supplement some missing data. Descriptive statistics of variables are shown in Table 1.

## 4. Empirical Design

### 4.1. Parallel Trend Test

**Figure 1:**
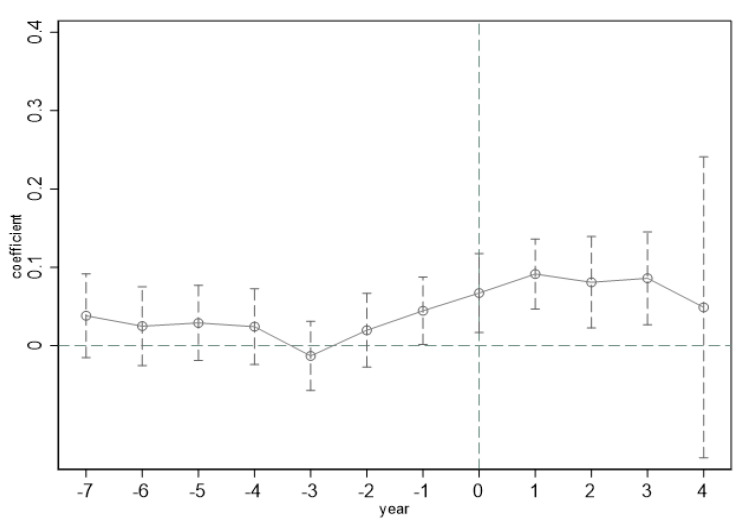
Parallel trend test results. Note: The *x*-axis represents the year before and after the construction of the NBDCPZ; −7~−1 represent years before NBDCPZ implementation; 0 represents the current year of policy implementation; and 1~4 represent years after policy implementation.

Drawing on the results of previous research, we conducted a common trend test on the selected samples [60]; the test results are shown in Figure 1. During the seven periods before the implementation of the NBDCPZ pilot policy, the coefficient of policy variables in each period has no significant difference from 0, indicating that the parallel trend assumption was met, that is, the policy effect evaluation of this study is applicable to the DID model. In the previous period of policy implementation, the current period, and the three subsequent periods, the policy coefficient is significantly greater than 0, and the policy coefficient is not significantly positive in the fourth period, preliminarily verifying that the construction of NBDCPZs has a significant positive impact on urban low-carbon transformation, which is first increased and then weakened, becoming insignificant. Possible reasons for this phenomenon include that in 2014, the Chinese government integrated “big data” into the government work report, and the document of the Platform for Action to Promote Big Data Development issued in 2015 directly include big data in China’s national strategy. The Chinese government has started to lay out the big data strategy as early as 2015. For some provinces and cities with the foundation of big data construction, local governments actively responded to national policies and plan ahead, which caused the policy effect of NBDCPZ occur ahead of time, in addition to supporting the implementation of the policy. Local governments should continue to implement the NBDCPZ policy in combination with their own development conditions. Pilot provinces and cities with poor NBDCPZ construction results should actively adjust and adopt an exit mechanism.

### 4.2. Bechmark Regression Results

Table 2 shows the benchmark regression results of the impact of NBDCPZ constructions on urban low-carbon transformation. In order to avoid multiple collinearity, leading to errors in the estimation results, we used the method of gradually adding control variables to improve the accuracy and reliability of the estimation results. Regardless of the model, NBDCPZ construction has a significant positive effect on urban low-carbon transformation and development. In model (9) in Table 2, compared with non-pilot cities, NBDCPZ construction can improve the low-carbon transformation of pilot cities by 0.0611%, indicating that the construction of NBDCPZs plays an important role in promoting the development of urban low-carbon transformation, verifying hypothesis H1.

### 4.3. Robustness Analysis

#### 4.3.1. Exclusion of Other Policy Pilots

The low-carbon transformation of cities may also be affected by the pilot policies of low-carbon cities, broadband cities, smart cities, innovative cities, and civilized cities in China. In order to investigate the “net effect” of NBDCPZ construction on urban low-carbon transformation and ensure the accuracy and reliability of the benchmark regression results, five policies were gradually incorporated into the benchmark regression model. Table 3 shows the model regression results. The coefficient of *Policy* is still significantly positive, indicating that after excluding the impact of other pilot policies in the same period and regions, NBDCPZ construction still has a significantly positive “net effect” on urban low-carbon transformation, implying that the benchmark regression results are reliable.

#### 4.3.2. Placebo Test

**Figure 2:**
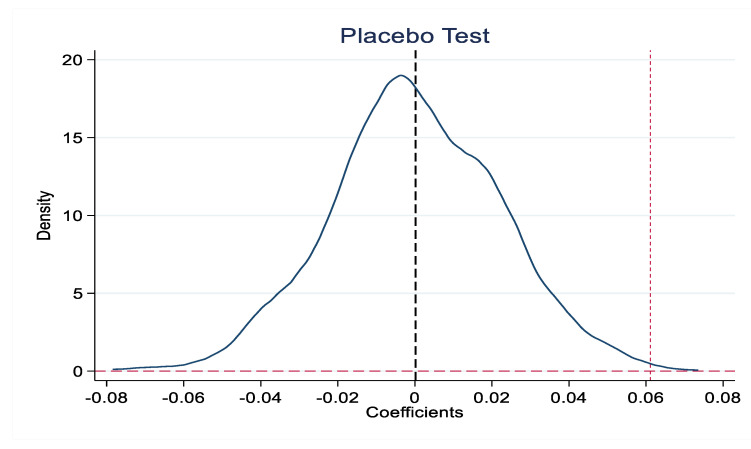
Placebo test.

In order to further exclude the influence of other random factors, drawing on existing research [61], we randomly selected the same number of cities as the control group and constructed a “virtual” *Policy* variable according to the number of pilot cities each year, repeating 1000 regressions on the benchmark model. The 1000 estimated coefficients of *Policy* variables were plotted as the kernel density distribution (Figure 2). Most of the simulated regression coefficient values are around 0, with a mean value of 0.0001, which is close to 0 relative to the estimated coefficient of *Policy* in the benchmark regression, indicating that the benchmark regression coefficient is greater than most analog values and can be considered an extreme value. Furthermore, the estimation results of the benchmark regression are not accidental. Therefore, the promotion effect of NBDCPZ construction on urban low-carbon transformation is not obviously affected by other random factors.

#### 4.3.3. Other Robustness Tests

We also conducted robustness tests considering the following six aspects. (1) In order to alleviate the subjectivity of the selection of pilot cities, we used propensity score matching combined with the difference-in-differences (PSM-DID) model to conduct robustness tests. (2) Assuming 2015 as the policy implementation year, we used the traditional DID method to re-estimate the effect of NBDCPZ construction on urban low-carbon transformation. (3) To delete the sample of cities with high administrative levels, such as municipalities directly under the central government and sub-provinces, the benchmark regression was conducted again. (4) The super-efficient SBM model was adopted to recalculate the urban carbon emission efficiency. (5) Drawing on existing research [62], an index system was used to measure the level of big data construction. (6) The carbon emission performance measured by carbon emission intensity (carbon emissions per unit GDP) was used as the explained variable for robustness tests. The results are shown in Table 4. The estimated coefficients of *Policy* in column (1) to column (5) were still significantly positive at the level of 1%. The coefficient of *Policy* in column (6) of Table 4 was significantly negative at the 10% level. These estimation results show the main finding that the positive effect exerted by NBDCPZ construction on urban low-carbon transformation is stable.

### 4.4. Conduction Mechanism Analysis

According to the theoretical mechanism analysis above, the construction of NBDCPZs can promote urban low-carbon transformation through conduction mechanisms, including the green innovation effect and resource allocation effect. Table 5 shows the regression results of the conduction mechanism test. Columns (1) and (2) in Table 5 show that the construction of NBDCPZs significantly promotes urban green innovation, and urban green innovation also significantly promotes urban low-carbon transformation. Compared with the benchmark regression coefficient of 0.0611, the estimated coefficient value of *Policy* in column (2) decreases, indicating that the construction of NBDCPZs can promote urban low-carbon transformation through urban green innovation, and urban green innovation plays a part of the intermediary effect. Columns (3) and (4) in Table 5 show that the construction of NBDCPZs has a promotion effect on the allocation of urban resource elements, and the allocation of urban resource elements also significantly promotes the low-carbon transformation of cities. Similarly, compared with the estimated coefficient of 0.0611 in the benchmark regression, the coefficient value of *Policy* in column (4) is decreased, indicating that the construction of NBDCPZs promotes the low-carbon transformation of cities through the allocation of urban resources, and the allocation of urban resources plays a role in the intermediary effect. This result implies that the green innovation effect and resource element allocation effect are important transmission channels for NBDCPZ construction to promote urban low-carbon transformation, verifying the hypothesis H2.

### 4.5. Impact Heterogeneity Analysis

In order to investigate the differential impact of NBDCPZ constructions on urban low-carbon transformation, we conducted an analysis from three perspectives: geographic location, urban scale, and urban natural resource endowment. First, we divided the samples into eastern, central, and western regions according to the geographic location of the city. Second, cities were divided into large, small, and medium-sized cities according to the population size. Third, cities were divided into resource-based cities and non-resource-based cities according to their natural resource endowment characteristics. The regression results are shown in Table 6. Compared with eastern cities, small and medium-sized cities, and resource-based cities, the construction of NBDCPZs can promote the low-carbon transformation of cities in the central and western regions, large cities, and non-resource-based cities, possibly because cities in the central and western regions have a broader development space. Before the implementation of the NBDCPZ pilot policy, the cities in the central and western regions were limited by the degree of infrastructure improvement, the scale of economic development, and other factors and were in the bottleneck stage of low-carbon transformation for a long time. The implementation of the NBDCPZ pilot policy broke the traditional extensive economic development model and solved the dilemma of low-carbon transformation construction in central and western cities. In addition, compared with small and medium-sized cities, large cities usually have advantages in terms of policy preferences, management authority, factor aggregation, etc. With the help of good institutional mechanisms and the market scale of big cities, NBDCPZ constructions can contribute to green innovation and resource allocation. Resource-based cities rely excessively on natural resources in terms of industrial structure and lack demand for innovative talents with digital technology; therefore, the low-carbon promotion effect of NBDCPZ construction is not always obvious.

## 5. Further Analysis

To further test the spatial spillover effect of NBDCPZ construction on urban low-carbon transformation, with referent to existing research [63,64,65], we used the difference-in-differences method combined with the spatial Durbin model (DID-SDM) based on the distance space weight matrix (1/*d*, where *d* is the linear distance of the city center) to investigate the spatial spillover effect of NBDCPZ construction. Table 7 shows the spatial effect decomposition results of the DID-SDM model. The construction of NBDCPZs obviously promotes the low-carbon transformation of local cities and significantly inhibits the low-carbon transformation of surrounding cities, indicating that the policy effect of NBDCPZ has significant spatial spillover characteristics. The hypothesis H3 is therefore verified, indicating that the negative spillover effect of NBDCPZ construction on the low-carbon transformation of surrounding cities is greater than the positive spillover effect, possibly because the construction of China’s NBDCPZs has occupied the resource elements of surrounding cities (policy pilot cities and non-policy pilot cities), and the surrounding cities excessively imitate local cities in big data construction, which is not conducive to the low-carbon transformation of surrounding cities.

## 6. Discussion

### 6.1. Conclusions

Under the dual background of global warming and the rise of the digital economy, data elements have become the core driving force for the digital economy to promote low-carbon economic transformation. Based on theoretical analysis, we considered the construction of NBDCPZ as a quasi-natural experiment and used China’s city-level panel data from 2007 to 2019 to thoroughly investigate the impact of big data construction on urban low-carbon transformation, drawing the following research conclusions. (1) According to the two-way fixed effect benchmark regression model based on the difference-in-differences (DID) method, it can be concluded that NBDCPZ construction can effectively promote urban low-carbon transformation. A series of robustness analysis supports this research conclusion. (2) The panel mediation effect model shows that stimulating urban green innovation and optimizing the allocation of urban resource elements are the main conduction mechanisms of NBDCPZ construction with respect to the promotion of urban low-carbon transformation. (3) The benchmark regression model was further tested and shows that compared with eastern cities, small and medium-sized cities, and resource-based cities, the construction of NBDCPZs can promote the low-carbon transformation of cities in central and western China, large cities, and non-resource-based cities. (4) According to the DID-SDM model, the construction of NBDCPZs can improve the low-carbon transformation of local cities, significantly inhibit the low-carbon transformation of surrounding cities, and exert a negative spatial spillover effect. Construction of NBDCPZs is one of the most fundamental measures for data elements and big data to highlight to the digital economy and promote the low-carbon transformation of the economy. In addition, “smart city” construction and “broadband China” construction are also important parts of China’s digital infrastructure construction. NBDCPZ construction, “smart city” constructions, and “broadband China” construction are mutually reinforcing and collaborative construction models. The conclusions of this study provide reference for further improvements to digital economic policies in China. For example, in the process of building “smart cities” and “broadband China”, the Chinese government should prevent them from exerting a negative impact on the low-carbon transformation of surrounding cities.

Based on the above research conclusions, the policy implications of this study are as follows. First, the Chinese government should continue to reasonably promote the orderly construction of NBDCPZs and highlight and promote the successful experience of the pilot cit. Second, the government should optimize the settlement policy of NBDCPZ digital talents, improve the software and hardware environment of NBDCPZ enterprise innovation, attach importance to the integration of data elements into traditional production, pay attention to the research and development and application of green and low-carbon technologies, and use big data technology to help the core technology breakthrough in the production field and the smart technology development of “imitation innovation”. In addition, it is necessary to constantly improve the property rights, pricing, and trading mechanisms of data elements. Policy makers should strive to build an integrated market system for NBDCPZ elements, break down institutional barriers that hinder the flow of elements, and use administrative means and market forces to continuously encourage the all-round complementation, coordination, and coupling of data resources and traditional resource elements so as to consolidate the external environment to encourage the green innovation effect and resource allocation effect of NBDCPZ construction. Thirdly, it is very important to strengthen cooperation, demonstration, imitation, and competition among various types of cities and to implement the construction of NBDCPZs according to local conditions. If necessary, eastern cities, large cities, and non-resource-based cities can expand pilots to the county level, and according to their own conditions, they can appropriately build a docking mechanism that is complementary to other pilot policies (broadband pilot policies, low-carbon pilot policies, etc.). Fourth, policy makers should carefully consider the negative spatial spillover effect of NBDCPZ construction, develop a cooperation and interaction mechanism between pilot cities, strengthen the learning and exchange mechanism between non-pilot cities and pilot cities, and minimize the negative spatial spillover effect of NBDCPZ construction.

### 6.2. Research Limitations and Future Directions

Although in this study, we systematically and thoroughly investigated the impact of NBDCPZ construction on urban low-carbon transformation, the following areas can still be improved. (1) In the future, the low-carbon effect of NBDCPZ construction can be considered at the enterprise level to investigate the impact of NBDCPZ construction on enterprise low-carbon transformation. (2) It is meaningful to combine the NBDCPZ pilot policy with other pilot policies and deepen the understanding of the impact of NBDCPZ construction on urban low-carbon transformation from the perspective of policy combination. (3) In the future, we will also focus on a certain type of ecological environment big data supervision platforms and carry out case studies. (4) Other future research directions include the use Python software to capture keywords related to data elements, the use of text analysis to build a data element index system, and accurately investigation of the impact of data elements on urban low-carbon transformation.

## Figures and Tables

**Table 1:** Descriptive statistics.

Variable	Symbol	Mean	N	S.D.	Min	P25	P50	P75	Max
Explained Variable	*Eff*	0.430	3679	0.270	0.030	0.250	0.350	0.510	1.640
Core Explanatory Variable	*Policy*	0.090	3679	0.280	0.000	0.000	0.000	0.000	1.000
Control Variable	*AGDP*	10.470	3679	0.780	8.130	9.950	10.420	10.940	13.190
	*URB*	0.450	3679	0.190	0.010	0.320	0.440	0.560	1.000
	*ER*	0.630	3679	0.190	0.060	0.490	0.680	0.770	0.990
	*AGG*	0.010	3679	0.030	0.000	0.000	0.010	0.010	0.460
	*OPEN*	1.820	3679	1.840	0.000	0.470	1.260	2.550	13.160
	*ROAD*	2.310	3679	0.590	−3.910	1.970	2.370	2.700	4.270
	*FIS*	0.380	3679	0.100	0.140	0.320	0.360	0.430	0.880
	*FIN*	0.900	3679	0.590	0.080	0.550	0.720	1.020	7.450
Mediating Variable	*INNOV*	0.540	3679	1.510	0.000	0.040	0.110	0.380	26.820
	*AE*	0.000	3679	0.030	−0.590	0.000	0.000	0.010	0.270

**Table 2:** Benchmark regression results.

Variable	(1)	(2)	(3)	(4)	(5)	(6)	(7)	(8)	(9)
*Policy*	0.0347 ***	0.0627 ***	0.0623 ***	0.0621 ***	0.0590 ***	0.0587 ***	0.0609 ***	0.0609 ***	0.0611 ***
	(2.625)	(4.556)	(4.519)	(4.505)	(4.306)	(4.280)	(4.454)	(4.447)	(4.460)
*AGDP*		0.1852 ***	0.1849 ***	0.1867 ***	0.1920 ***	0.1924 ***	0.1886 ***	0.1900 ***	0.1863 ***
		(8.261)	(8.266)	(8.347)	(8.612)	(8.558)	(8.415)	(8.141)	(7.338)
*URB*			0.0775 *	0.0764 *	0.0843 *	0.0841 *	0.0841 *	0.0843 *	0.0836 *
			(1.756)	(1.721)	(1.935)	(1.933)	(1.936)	(1.939)	(1.920)
*ER*				0.0439	0.0507 *	0.0509 *	0.0567 **	0.0570 **	0.0576 **
				(1.579)	(1.819)	(1.826)	(2.023)	(2.029)	(2.052)
*AGG*					1.5542 ***	1.5456 ***	1.6225 ***	1.6164 ***	1.6071 ***
					(4.392)	(4.366)	(4.533)	(4.520)	(4.487)
*OPEN*						−0.0007	−0.0015	−0.0015	−0.0017
						(−0.269)	(−0.547)	(−0.537)	(−0.591)
*ROAD*							0.0383 ***	0.0383 ***	0.0386 ***
							(2.678)	(2.683)	(2.706)
*FIS*								−0.0273	−0.0231
								(−0.271)	(−0.229)
*FIN*									−0.0064
									(−0.511)
*Constant*	0.4238 ***	−1.5168 ***	−1.5492 ***	−1.5948 ***	−1.6786 ***	−1.6813 ***	−1.7337 ***	−1.7377 ***	−1.6952 ***
	(148.386)	(−6.452)	(−6.564)	(−6.720)	(−7.090)	(−7.064)	(−7.262)	(−7.226)	(−6.367)
*N*	3679	3679	3679	3679	3679	3679	3679	3679	3679
*Fixed effects*	Yes	Yes	Yes	Yes	Yes	Yes	Yes	Yes	Yes
*R* ^2^	0.6669	0.6740	0.6743	0.6746	0.6769	0.6769	0.6778	0.6778	0.6778
*Adj-R* ^2^	0.6379	0.6455	0.6457	0.6459	0.6483	0.6482	0.6490	0.6489	0.6489
*F*	6.8908	35.4580	24.5632	19.2104	19.7909	16.5996	15.1362	13.3298	12.2664

Note: the numbers in parentheses are robust t-statistics. ***, **, and * represent significance levels of 1%, 5%, and 10%, respectively.

**Table 3:** Exclusion of other policy pilots.

Variable	(1)	(2)	(3)	(4)	(5)
*Policy*	0.0607 ***	0.0630 ***	0.0645 ***	0.0657 ***	0.0688 ***
	(4.426)	(4.612)	(4.720)	(4.805)	(5.015)
*Low-carbon city pilots*	0.0161	0.0144	0.0142	0.0118	0.0112
	(1.422)	(1.277)	(1.264)	(1.051)	(0.997)
*Broadband city pilots*		0.0471 ***	0.0451 ***	0.0399 ***	0.0349 ***
		(4.637)	(4.469)	(3.964)	(3.482)
*Smart city pilots*			0.0210 **	0.0178 *	0.0131
			(2.161)	(1.839)	(1.354)
*Innovative city pilots*				0.0650 ***	0.0555 ***
				(4.995)	(4.248)
*Civilized city pilots*					0.0530 ***
					(4.983)
*Control variables*	Yes	Yes	Yes	Yes	Yes
*Constant*	−1.6724 ***	−1.6601 ***	−1.6388 ***	−1.6049 ***	−1.5973 ***
	(−6.251)	(−6.212)	(−6.107)	(−5.975)	(−5.927)
*N*	3679	3679	3679	3679	3679
*Fixed effects*	Yes	Yes	Yes	Yes	Yes
*R* ^2^	0.6780	0.6798	0.6801	0.6820	0.6839
*Adj-R* ^2^	0.6490	0.6508	0.6511	0.6530	0.6550
*F*	11.4160	12.5568	12.1451	12.7738	13.2974

Note: the numbers in parentheses are robust t-statistics. ***, **, and * represent significance levels of 1%, 5%, and 10%, respectively.

**Table 4:** Other robustness tests.

Variable	(1)	(2)	(3)	(4)	(5)	(6)
	PSM-DID	Traditional DID	Reselect Samples	*Eff* Estimated by SMB Method	Big Data Construction Estimated by an Index System	Carbon Emission Intensity Used to Measure the Explained Variable
*Policy*	0.0614 ***	0.0628 ***	0.0551 ***	0.0599 ***	0.6411 ***	−0.2655 *
	(4.431)	(4.431)	(3.868)	(4.062)	(3.263)	(−1.660)
*Controls*	Yes	Yes	Yes	Yes	Yes	Yes
*Constant*	−1.6972 ***	−1.7640 ***	−1.5672 ***	−1.9779 ***	−2.2609 ***	−1.0763
	(−6.077)	(−6.462)	(−5.759)	(−7.071)	(−6.857)	(−0.269)
*N*	3656	3627	3432	3679	2546	3679
*R* ^2^	0.6750	0.6798	0.6739	0.6780	0.7198	0.3735
*Adj-R* ^2^	0.6456	0.6510	0.6445	0.6491	0.6825	0.3172
*F*	11.8121	12.3995	10.1158	13.0036	15.6316	3.2519

Note: the numbers in parentheses are robust t-statistics. ***, and * represent significance levels of 1%, and 10%, respectively.

**Table 5:** Regression results of conduction mechanism test.

Variable	(1)	(2)	(3)	(4)
*Policy*	0.2115 **	0.0553 ***	0.0057 ***	0.0587 ***
	(2.169)	(4.075)	(2.631)	(4.279)
*INNOV*		0.0277 ***		
		(6.037)		
*AE*				0.4264 ***
				(2.999)
*Controls*	Yes	Yes	Yes	Yes
*Constant*	−3.4499 ***	−1.5996 ***	0.0528	−1.7178 ***
	(−3.039)	(−5.945)	(1.004)	(−6.462)
*N*	3679	3679	3679	3679
*Fixed effect*	Yes	Yes	Yes	Yes
*R* ^2^	0.7858	0.6831	0.2366	0.6791
*Adj-R* ^2^	0.7666	0.6546	0.1680	0.6502
*F*	21.8620	14.4079	2.2701	11.8988

Note: the numbers in parentheses are robust t-statistics. ***, and ** represent significance levels of 1%, and 5%, respectively.

**Table 6:** Impact heterogeneity discussion.

Variable	Location		Population Scale		Resource Endowment	
	Eastern	Central and Western	Large Scale	Medium and Small Scale	Resource-Based Cities	Non-Resource-Based Cities
*Policy*	0.0388 *	0.0898 ***	0.0810 ***	0.0455 **	0.0228	0.0772 ***
	(1.769)	(5.208)	(4.615)	(2.288)	(1.137)	(4.334)
*Controls*	Yes	Yes	Yes	Yes	Yes	Yes
*Constant*	−2.8205 ***	−1.3901 ***	−2.3544 ***	−1.1072 ***	−1.1551 ***	−1.7573 ***
	(−6.390)	(−3.749)	(−7.845)	(−2.844)	(−2.946)	(−4.794)
*N*	1547	2132	1820	1859	1482	2197
*Fixed effects*	Yes	Yes	Yes	Yes	Yes	Yes
*R* ^2^	0.6135	0.7262	0.7080	0.6712	0.6756	0.6890
*Adj-R* ^2^	0.5753	0.7003	0.6798	0.6395	0.6434	0.6597
*F*	12.4594	8.6960	16.2446	12.2253	6.3532	11.6126

Note: the numbers in parentheses are robust t-statistics. ***, **, and * represent significance levels of 1%, 5%, and 10%, respectively.

**Table 7:** Spatial effect decomposition results of the DID-SDM model.

Variable	*LR_Direct*	*LR_Indirect*	*LR_Total*
*Policy*	0.0834 ***	−0.3937 **	−0.3102 **
	(4.555)	(−2.400)	(−2.019)
*Controls*	Yes	Yes	Yes
*Time fixed effect*	Yes	Yes	Yes
*Spatial fixed effect*	Yes	Yes	Yes
*rho*	0.3473 ***		
	(3.090)		
*Sigma2_e*	0.0225 ***		
	(42.847)		
*N*	3679		
*R* ^2^	0.0086		
*Log likelihood*	1754.1226		

Note: the numbers in parentheses are robust t-statistics. ***, and ** represent significance levels of 1%, and 5%, respectively.

## Data Availability

The original data on variables are from *China Urban Statistical Yearbook* (2008–2020), the *China Energy Statistical Yearbook* (2008–2020), provincial and municipal statistical yearbooks, and the EPS data platform.
